# Metagenomic Predictions: A Review 10 years on

**DOI:** 10.3389/fgene.2022.865765

**Published:** 2022-07-20

**Authors:** Elizabeth M Ross, Ben J Hayes

**Affiliations:** Centre for Animal Science, Queensland Alliance for Agriculture and Food Innovation, University of Queensland, Brisbane, QLD, Australia

**Keywords:** metagenomics, microbiome, prediction, methane, feed efficiency

## Abstract

Metagenomic predictions use variation in the metagenome (microbiome profile) to predict the unknown phenotype of the associated host. Metagenomic predictions were first developed 10 years ago, where they were used to predict which cattle would produce high or low levels of enteric methane. Since then, the approach has been applied to several traits and species including residual feed intake in cattle, and carcass traits, body mass index and disease state in pigs. Additionally, the method has been extended to include predictions based on other multi-dimensional data such as the metabolome, as well to combine genomic and metagenomic information. While there is still substantial optimisation required, the use of metagenomic predictions is expanding as DNA sequencing costs continue to fall and shows great promise particularly for traits heavily influenced by the microbiome such as feed efficiency and methane emissions.

## Introduction

**FIGURE 1:**
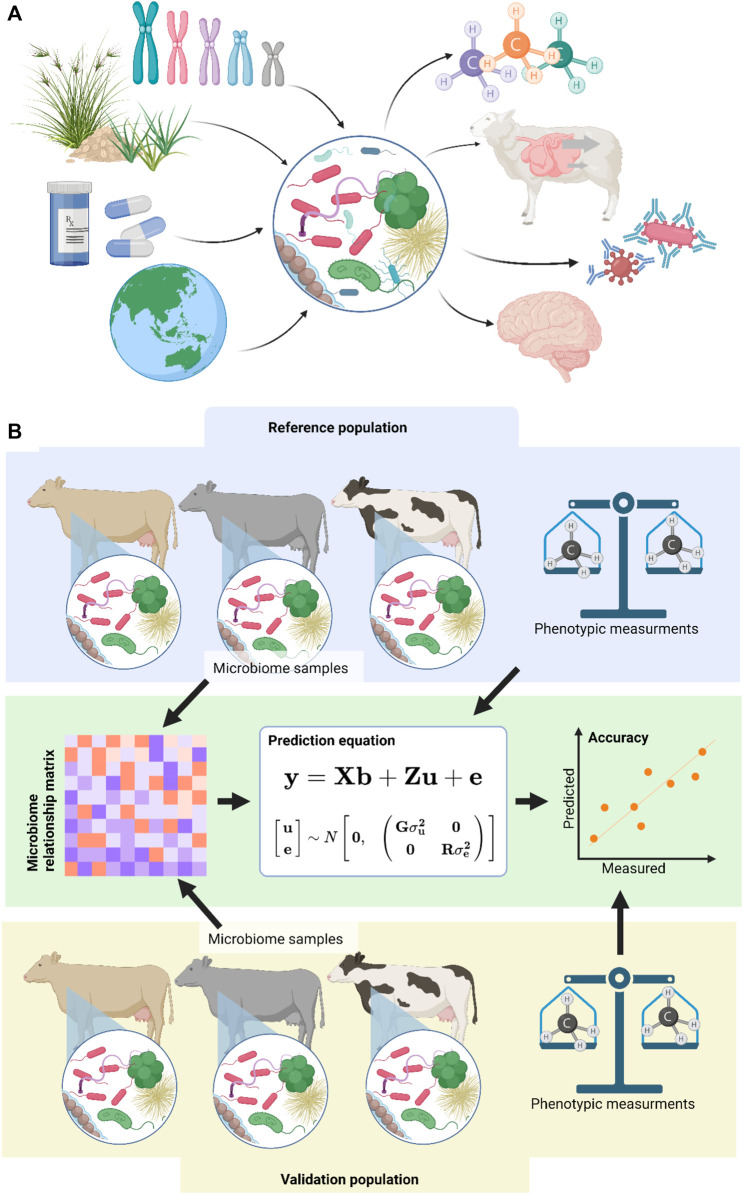
**(A)** The host associated microbiome is influenced by a range of factors including host genetics, diet, drugs and medication, and physical location. In turn the host associated microbiome is thought to influence several phenotypes including enteric methane production, feed conversion efficiency, immune function, and even neurological traits. **(B)** Metagenomic predictions use a reference population of microbiome samples and measured phenotypes to predict unknown phenotypes in a difference validation population with only microbiome samples. The accuracy of the prediction can be evaluated by comparing measured phenotypes in the validation population to these predicted by the model. Image generated in BioRender.

The host associated microbiome is known to influence many traits (Figure 1A), including health traits (Cho and Blaser, 2012; Rothschild et al., 2022), enteric methane production (Ross et al., 2013b; Wallace et al., 2015; Hess et al., 2020; Hess et al., 2021), feed efficiency (Wang et al., 2015; Wen et al., 2021), carcass traits (Maltecca et al., 2019) and even neurological traits (Kho and Lal, 2018). The metagenome is the cumulative genomes of the cells which make up the microbiome. Metagenomics is the study of that genome population. Metagenomic predictions use the variation in metagenomes to predict the phenotype of a host (Ross et al., 2013b). While the exact mechanisms through which microbiomes effect the host phenotype are not always known, some direct effects, such as methanogens producing methane (Morgavi et al., 2010), and some indirect effects, such as the modulation of the host immune system (Levy et al., 2017), have been identified in various species. While metagenomic predictions rely on these underlying causative effects, knowledge of them is not required for accurate metagenomic predictions, as the relationships calculated are purely mathematical, and currently do not consider biological relationships.

This brief review examines work done to explore the predictive potential of the host associated microbiome, with a focus on ruminant livestock traits.

## Phenotypic Traits

The metagenomic prediction method (Figure 1B) was originally inspired by genomic prediction, which use relationships between samples derived from DNA marker genotypes to predict unknown phenotypes using best linear unbiased prediction (BLUP). Metagenomic predictions were first reported in 2012 (Ross et al., 2012b) where they were used to predict sample type and inflammatory bowel disease status. Soon after, they were used to predict methane production levels from cattle (Ross et al., 2013b; Table 1), which has since been replicated in sheep (Ross et al., 2020; Hess et al., 2021).

**TABLE 1:** Summary of studies which have used rumen metagenomic profiles to predict phenotypes in ruminants.

Study	Species (N^#^)	Phenotype	Method	Within or between Countries	Accuracy	
					Microbiome Only	Microbiome and Genome
Ross et al. (2013a)	Dairy cattle (62)	Enteric methane	Co-variance matrix and BLUP	Within	<0^NS^–0.79	-
			Random Forests	Within	0.33	-
Wang et al. (2015)	Dairy cattle (28)	Residual feed intake	Co-variance matrix and BLUP	Within	0.08^NS^ - 0.49	0.38–0.57
Delgado et al. (2019)	Dairy cattle (61)	Feed efficiency	Linear effects	Between	0.19	-
		Dry matter intake	Linear effects	Between	0.39	-
Ross et al. (2020)	Sheep (99)	Enteric methane	Co-variance matrix and BLUP with microbiome	Within	<0^NS^–0.14	0^NS^–0.25
			Co-variance matrix and BLUP with metabolome	Within	0.13–0.25	0.16–0.27
Hess et al. (2020)	Sheep (340)	Enteric methane	Principle component analysis	Within	0.17–0.51*	-
Hess et al. (2021)	Sheep (1702)	Enteric methane	Correlation matrix and BLUP	Between	<0^NS^ - 0.13^NS^	<0^NS^ - 0.13^NS^
			Correlation matrix and BLUP	Within	0.40–0.57	0.53–0.60

# Number of animals used in the entire study, including both reference and validation populations.

* Not cross validated.

NS Not significantly different to 0.

Metagenomic predictions were subsequently used to predict residual feed intake (Wang et al., 2015). In chickens metagenomic variation of the caecum was found to be associated with residual feed intake, but not other gut locations (Wen et al., 2021). Carcass traits in pigs have been predicted with moderate to high accuracy from gut microbiomes by Maltecca et al. (2019). Recently, research in sheep used metagenomic predictions to predict methane yield in sheep in Australia (Ross et al., 2020) and in New Zealand (Hess et al., 2021). Additional studies have found further associations between microbiome variation and methane in cattle (Difford et al., 2018; Zhang et al., 2020; Andrade et al., 2022).

Methane and residual feed intake are expected to have a direct link to the gut metagenome composition, and hence a relationship between metagenome variation and phenotypic variation for these traits might be expected. On the other hand, a recent study in sheep found that the metagenome did not explain any of the phenotypic variance of dairy traits in sheep (Martinez Boggio et al., 2022), despite some rumen bacteria being associated with milk characteristics (Martinez Boggio et al., 2021). Conversely, Gebreyesus et al. (2020) found that the rumen metagenome was predictive of ketone bodies in milk, an indicator of ketosis, which has previously been associated with rumen microbiome changes (Zhu et al., 2018). This may suggest that either the link between the phenotype of interest and the microbiome needs to be particularly strong for metagenomic predictions to work, or that more sophisticated models are required.

Microbiome associated traits have also been predicted in humans. Body mass index was predicted within and across populations from gut samples (Ross et al., 2013b; Rothschild et al., 2022), as was Crohn’s disease (Asgari et al., 2018) and ulcerative colitis (Ross et al., 2013b). Four other health related traits including menopausal status and smoking status were linked to skin microbiome variation in Carrieri et al. (2021), while Rothschild et al. (2022) predicted a number of traits including smoking status and type II diabetes. Overall, it is apparent that microbiome variation is associated with a large range of host phenotype traits, although the exact causal relationship is not always clear-cut.

## The Counts Matrix

**FIGURE 2:**
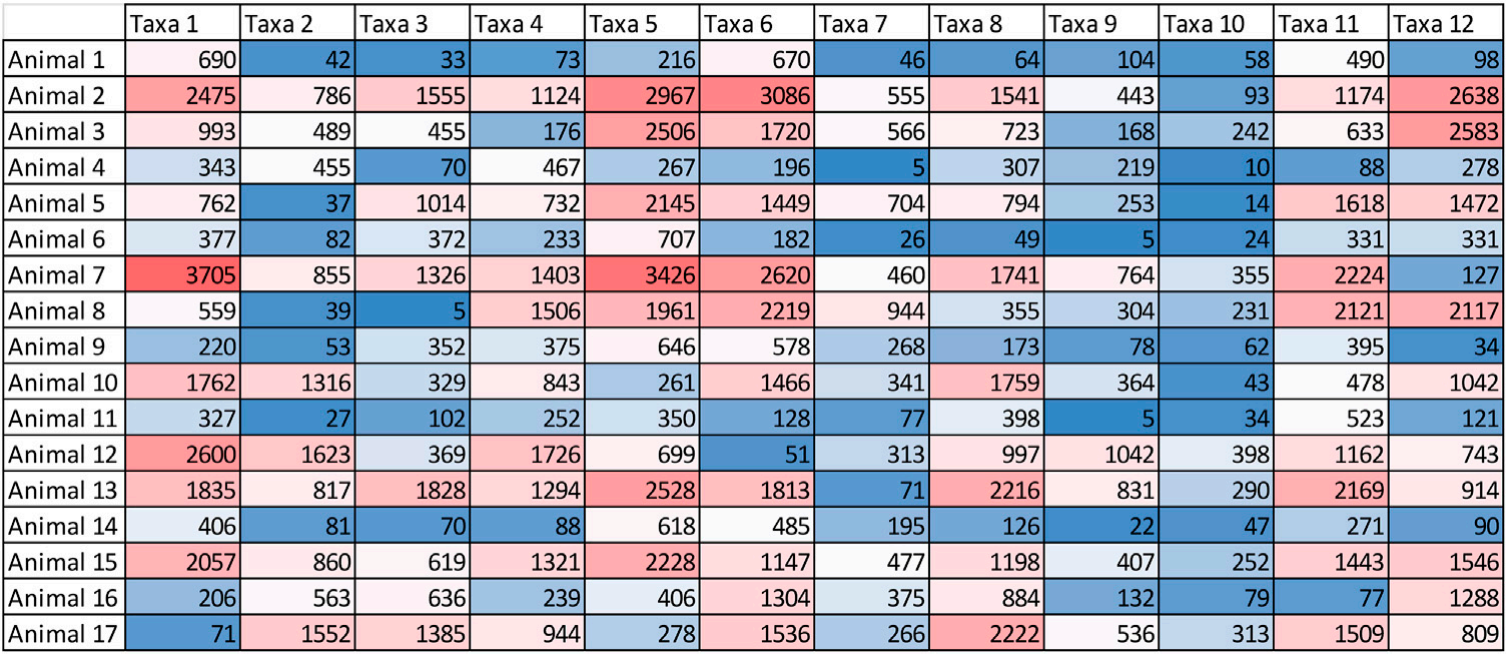
An example of the count matrix that can be used to capture the variation in the microbial population. Note that the total number of reads for each animal may vary, as in this example, and should be standardised. While this example only includes 12 taxa, many thousands of taxa can be included in the count matrix.

All metagenomic predictions begin with a “counts matrix” which attempts to approximate the proportion of different taxa in each animal’s microbiome (Figure 2). This is challenging given many of the species in the microbiome are unknown. Originally, metagenomic predictions used short read shotgun sequences aligned to a reference genome of microbial species (or sequence assemblies) to approximate the proportion of different taxa (Ross et al., 2013b). This approach should be more accurate, for cattle at least, now that a good proportion of the rumen microbes have been fully sequenced (e.g., Seshadri et al., 2018).

Methods which use 16S sequencing and reduced representation sequencing have also been successfully used in metagenome predictions (Hess et al., 2020; Ross et al., 2020), although if a species is not represented in the 16S database, or not captured by the selected primer, it will not appear in the counts matrix. Another approach, used by Maltecca et al. (2019), aligned sequence reads to operational taxonomic units (OTUs). Other methods that provide the ability to classify reads such that they represent some sort of taxonomical groupings could equally be implemented for the generation of the relationship matrix, such as amplicon sequence variants (Callahan et al., 2017), or any other method that achieves the same end point of a count matrix which is able to capture relative changes of microbial abundances.

Once the count matrix that represents different microbial species abundance is formed, a co-variance matrix was calculated from the count matrix which was used to predict phenotypes in a non-overlapping group of individuals. The methods above were the starting point for metagenomic predictions, but future work will likely find that there are more optimal approaches. For example, as more and more rumen microbes have their genomes completely sequenced, alignment at a species level becomes possible.

## Metagenomic Relationship Matrix Calculation Methods

The original metagenomic prediction method used a relationship co-variance matrix among animals that was calculated as **XX**’/m, where **X** is the (standardised) count matrix described above and m is the number of contigs or taxa used to make the count matrix. Subsequently (Hess et al., 2020; Hess et al., 2021) used a correlation matrix to overcome convergence issues. Earlier work (Ross et al., 2012a) used the Canberra method (Lance and Williams, 1966) to generate a distance matrix. Other distance methods should be explored in the larger emerging datasets to optimise the representation of microbiome similarities, and improve convergence of the models.

The relationship matrix can be used in a best linear unbiased prediction approach (BLUP) to predict trait performance for individual animals. If the relationship matrix is appropriately standardised, The BLUP methods assume rare species contribute equally to the relationship matrix as highly abundant species.

The BLUP method begins with fitting a linear mixed model to the data:

y=Wβ+Zu+e

Where **y** is vector of phenotypic records, **W** is a design matrix allocating records to fixed effects such as sex, age, cohort, **β** are the values for these fixed effects, **Z** is a design matrix allocating records to individuals, and **e** is a vector of random errors. The **u** are random effects for individuals assumed distributed $N\left(0,\mathrm{MRM}\sigma_{m}^{2}\right)$ , where **MRM** is the metagenome relationship matrix, and $\sigma_{m}^{2}$ is the variance in the trait associated with the metagenome. Note that with model, and an appropriate experimental design, the effects of sex, age, cohort and so on can be disentangled from metagenome effects. This equation can be solved for metagenome predictions $\left(\hat{u}\right)$ for each individual using BLUP, and also for the effect of each individual species/OTU/contig in the metagenome as

$$\hat{g}=\mathrm{X\prime MR}M^{-1}\hat{u}/m$$

While the effects of differential weighting based on abundance have not been explored, one hypothesis could be that similarities within the metagenomic relationship matrix should be weighted by species abundance. This would also reduce the effect of random variation in the less abundant species affecting the observed relationships, especially with the low sequencing depth that is required for larger scale use of metagenomic predictions of phenotypes.

Something that is not easily captured by the co-variance relationship model is non-linear effects. Some machine learning methods can capture non-linear effects, however the number of samples required is large since the effect distribution needs to be estimated from the data. In genomic predictions of complex genomes non-linear effects can be captured by manipulation of the relationship matrix to represent interactions and non-linear patterns, for example a an organism with low abundance might have an effect with small changes in abundance, whereas an organism with high abundance might only have an effect with large changes in abundance. Including non-linear effects have resulted in an increased accuracy for some traits in genomic prediction (Yadav et al., 2021). A similar approach could be applied to microbiome samples to overcome this limitation, however the proportion of non-linear effects, and whether they in fact matter at all, is currently unknown.

A limitation of the relationship matrix-based prediction approach is that all metagenome species are assumed to have a small, but non-zero effect on the trait. . An advantage of relationship-based models is that they are feasible with smaller datasets, as thousands of effects do not need to be estimated from the data. As sequence generation costs continue to fall, the limitation of sample sizes may soon be resolved and non-relationship matrix-based prediction methods that are able to place more emphasis on associated species may prove more appropriate for metagenomic predictions.

## Prediction Methods

The limitations for relationship matrix-based predictions bring us to the next logical step–the use of prediction models that allow different weightings on different features, including the possibility of zero effect. In genomic predictions, given sufficient reference set size, Bayesian prediction models such as BayesR outperform relationship-based models. The first step toward using such methods in metagenome predictions this has already been completed by Zhang et al. (2020) who used Bayesian methods to examine methane variance explained by the metagenome in dairy cattle. In genomic prediction, methods such as BayesRC have been used to include biological priors to increase the accuracy of prediction further from SNP data (Macleod et al., 2016). These methods could be directly applied to large metagenomic datasets to allow species that are known to be associated with the trait of interest to be more highly weighted in the model. For example, taxa in studies that have been correlated with the target trait, such as the ∼500 taxa that Delgado et al. (2019) used for prediction, could be treated as a separate class in a BayesRC type approach.

Another option would be to increase the weighting (or treat as a separate class in BayesRC) species which contain the genes that are used in the relevant biological pathway. For example, for the prediction of enteric methane production, species which contain the methanogenesis pathway, or alternate hydrogen sinks (for examples see Morgavi et al., 2010) such as propionate formation, could be given larger priors in the same manner that SNP with biological priors can be more heavily weighted in some Bayesian prediction models. These taxa could be identified by mining the genome ontology terms in the fully sequenced rumen bacterial genome assemblies or the metagenome associated genomes.

A number of studies have proposed the use of machine learning for phenotypic prediction from metagenomes, mostly in humans and in pigs (e.g., Maltecca et al., 2019). These methods were recently reviewed by Marcos-Zambrano et al. (2021). At least in pigs, machine learning approaches such as random forest and gradient boosting, gave similar accuracies of prediction as BLUP. Some other recent examples of the use of machine learning to predict phenotypes from the microbiome include Asgari et al. (2018); Lo and Marculescu (2019); Fukui et al. (2020); and Carrieri et al. (2021). Carrieri et al. (2021) used the skin microbiome to predict a range of phenotypes in humans by applying explainable artificial intelligence. Reflux disorders were predicted by Lo and Marculescu (2019), and inflammatory bowel disease was a mutual target of a number of studies (Asgari et al., 2018; Lo and Marculescu, 2019; Fukui et al., 2020). These approaches illustrate the power of machine learning for phenotypic prediction when the dataset is large, however a direct comparison of these methods with BLUP based predictions has not been well examined. Ross et al. (2013b) compared BLUP and random forests in the same dataset, with BLUP outperforming random forests in both animal and human associated microbiomes. Rothschild et al. (2022) compared ridge regression-based predictions to gradient boosted decision trees, and found that the two methods were mostly comparable, but gradient boosted decision trees outperformed the regression on binary traits. Given the computational expense of machine learning, a significant benefit in terms of prediction accuracy would be required to justify their use over more basic methods, which is likely dependant on sample size.

## Combining Metagenomic and Other Prediction Systems

A handful of studies have examined the effect of combining genomic and metagenomic predictions. The first study to do so was Wang et al. (2015) who combined SNP data and metagenomic data in a small study to predict residual feed intake with higher accuracy than either method alone. While not used for metagenomic predictions, Difford et al. (2018) found that the variance explained by both the genomic and metagenomic data for dairy cows was greater than when either one was examined alone, as did Zhang et al. (2020) on the same dataset using a Bayesian method. Saborío-Montero et al. (2021) concluded that not only are both the genome and metagenome important for explaining the phenotypic variation, but that the interaction between genome and metagenome is also important. Recently, Ross et al. (2020) combined metagenomic and genomic predictions for studying enteric methane production in sheep. The study had a limited number of biological replicates (*N* = 99) but illustrated a proof of principle that the accuracy of the phenotypic prediction of enteric methane production was increased when metagenomic, or metabolomic, predictions from the rumen were included. Subsequently this finding was validated in a much larger cohort of animals (*N* = 1702) by Hess et al. (2021). Recently in pigs (Aliakbari et al., 2022) the accuracy of prediction for a number of traits including residual feed intake and back fat depth were shown to increase when both genetic and microbiome information was used in the prediction model.

The metagenome itself has some heritable components (Wallace et al., 2019; Abbas et al., 2020; Grieneisen et al., 2021; Cardinale and Kadarmideen, 2022), with heritabilities of individual genera up to 0.59 (Martínez-Álvaro et al., 2022). Therefore, there is expected to be overlap when selecting either based on metagenomic and genomic prediction values. The extent of host-metagenome interaction is important to consider when metagenomic predictions are used in selection. If there is no host genome -metagenome interaction, such selection may shift the current population mean (e g., through culling), but will not result in genetic improvement Conversely, if host-metagenome interaction is extensive, selection on metagenome predictions will result in genetic gain. Host genome -metagenome interaction could also explain why the accuracy of genomic and metagenomic predictions is not fully additive, that is, they are not detecting independent factors.

The additional accuracy observed when combining genomic and metagenomic information of the phenotype prediction accuracy is probably partially due to the metagenomic predictions capturing part of the environmental variation. The environmental variation is, by definition, not captured by genomic predictions. It is important to understand the interactions between different genomic and metagenomic predictions of a trait. Each trait is likely to have a unique profile of genomic, metagenomics and uncaptured environmental variation, that needs to be understood through experimentation. Key to this understanding is that unlike in genomic predictions where the aim is to predict the heritable component of the phenotype, metagenomic predictions usually aim to predict the phenotype itself.

## Prediction Pitfalls–Cause and Effect

A careful interpretation of the values generated by metagenomic predictions is needed. Critically, as opposed to genomic predictions, the effect direction of metagenomic predictions is not necessarily known. That is: is the microbiome affecting the phenotype, or is the phenotype affecting the microbiome? In the case of enteric methane production, it is most likely that the microbiome is affecting the phenotype, as there is not a documented mechanism for methane concentration to affect microbiome composition, but the mechanism for microbiomes to affect methane production is well understood. Not all traits are so clear-cut.

We can take lessons in the directionality of the metagenome’s effects not only from livestock research, but also from human and medical research. A recent study has illustrated the pitfalls of assuming that microbiome differences are causing host phenotype variation in humans. Yap et al. (2021) examined the link between autism spectrum disorder, diet, and the microbiome. They concluded that although variation in the microbiome is associated with autism spectrum disorder, it is not the cause. Rather, dietary preferences and limitations that are caused by autism spectrum disorder affect the microbiome composition. Therefore, it is the phenotype affecting the microbiome, not the other way around. Understanding this is critical for the correct use of metagenomic predictions, where the misuse of the information, such as attempting to alter the microbiome to reduce symptoms associated with autism spectrum disorder would be detrimental to the patients without any benefit.

## Population Differences

Another challenge facing the use of metagenomic predictions is the effect of environment and location. Hess et al. (2021) showed that sheep from New Zealand could not be used to predict methane in sheep from Australia, but that within country predictions were successful. Conversely, Delgado et al. (2019) used a Spanish dairy herd to predict the feed efficiency of an Australian dairy herd with success. A cause behind the phenomena that geographically separate populations may show poorer prediction accuracy than expected given their relationships in the relationship matrix is that there may be strain level differences in the species that make up the microbiome. Different geographical regions may have strains of bacteria that carry a different subset of genes compared to those found in other locations. This could result in a breakdown between the association with the trait in the reference population, and the prediction ability in the validation population because the sequence that is being quantified is not connected to the same causal gene in both populations.

There is also the possibility that not all causal agents exist in all populations. This is equivalent to having fixed alleles in genomic predictions, where there is no variation in the genome/metagenome at that position in the discovery population, and so it is not used in the prediction even if it is present in the validation population. Where low across-population prediction accuracies are observed it may be that it is only possible to overcome this hurdle by the inclusion of phenotyped individuals from the same location as the target population.

## Measuring Accuracy and Microbiability

Prediction accuracies for metagenomic predictions of continuous traits are generally reported as the Pearson’s correlation (r) between the predicted and the observed phenotype of the validation set for continuous traits. This is opposed to genomic predictions where *r* is scaled by the heritability of the trait by dividing by the square root of the narrow sense heritability. Analogous to the heritability is the microbiability. The microbiability is the proportion of the variance in the phenotype that can be attributed to the metagenomic relationship matrix. The microbiability however has substantial limitations including that it does not capture non-additive relationships, as pointed out by Rothschild et al. (2022).

The microbiability varies considerably across traits, for reasons described above. For example Aliakbari et al. (2022) gave estimates of 0.11 residual feed intake, 0.20 feed conversion ratio, and 0.02  backfat in pigs, while He et al. (2022) reported 0.42 for back fat. Hess et al. (2020) used both 16S sequencing, and reduced representation using restriction enzyme sequencing to calculate the microbiability for methane emission level. They revealed two things: that there is a substantial difference in the microbiability of the same dataset based on whether the data was derived from 16S or reduced representation sequencing; and also that the restriction enzyme chosen for reduced representation sequencing had a large impact on the microbiability. Saborío-Montero et al. (2021) found that the method used to calculate the microbiome relationships also affects the microbiability. This would suggest that the microbiability is strongly reflective of the method used and thus any comparison of microbiability between studies should be done with extreme caution. It also suggests that this measure could be a useful tool to compare methods.

## Parameters Affecting Accuracy of Metagenome Predictions

The microbiability is one parameter affecting the accuracy of metagenomic predictions–the higher this is (the greater proportion of the phenotypic variance captured by microbiome variation), the higher the accuracy of prediction.

Sample size is another key parameter. Prediction methods are limited by the number of samples that are available for use. The first metagenomic predictions had very limited biological replicates available and far too few to estimate effects for each species individually. Sequencing costs have plummeted as technology has advanced, and new methods of metagenomic profiling have become available (e.g., Hess et al., 2020). Thus, the limitation on sample numbers has moved from the sequencing cost to the cost of phenotyping, especially for traits that are expensive or difficult to measure such as enteric methane production. One exception is the study of Rothschild et al. (2022) where more than 30,000 samples were used in metagenomic predictions. That study demonstrated that accuracy plateaued with approximately 4,000 samples for most traits. For example, for BMI, the r value (the square root of the reported *r* ^2^) was approximately 0.32 for 2000 samples, 0.36 at 4,000 samples, and 0.38 for 8,000 samples. Thus, although increases in accuracy continue to be observed, there are diminishing returns as the sample same increases.

Building on theory that was developed to deterministically predict the accuracy of genomic selection with BLUP models (Daetwyler et al., 2008; Hayes et al., 2009), we would expect the accuracy of metagenome predictions to be, for BLUP predictions at least:

$$r=\sqrt{\frac{N_{P}m^{2}}{N_{P}m^{2}+P}}$$

Where *N*_*p*_ is the number of samples with phenotypes, *m* ^*2*^ is the microbiability, and *P* is the number of independent entities in the microbiome population. Approximations of *P* could be the number of OTUs, or the number of principal components required to capture >99% of the variance in the metagenome relationship matrix described above (e.g., Kittelmann et al., 2014).

In deriving the accuracy of metagenomic predictions, it is important to note that the microbiability changes with time. For example, Maltecca et al. (2019) found that the accuracy of metagenomic predictions for backfat (in pigs at 22 weeks of age) from samples at weaning were lower than from samples taken at the same time as phenotyping. For example, accuracy of prediction of back fat increased from *r* = 0.42 when microbiome samples from weaning to *r* = 0.48 when microbiome samples were taken at week 22.

## Utility of Metagenomic Predictions

The most basic use of metagenomics is the direct inference of the phenotype. Such direct inference could be used for direct selection, or diagnosis for intervention. For example, metagenomic predictions could be used to identify and remove high methane emitting cattle from a herd to lower a producer’s overall carbon footprint. Metagenomic predictions could also be used to select breeding animals with favourable traits such as high feed conversion efficiency (provided there was considerable host genome–metagenome interaction), or to diagnose conditions which may cause a shift in rumen ecology, such as sub-acute acidosis.

The microbiome could also be used for genomic selection in the future by generating proxy traits. Proxy traits are traits which approximate the true trait of interest. For example, metagenomic predictions could be used to generate predicted methane emission levels for large numbers of cattle. Those cattle could then be genotyped and genomic estimated breeding values for metagenomic-methane proxy traits could be calculated. Selection pressure could then be applied through breeding from the most desirable animals. Given that there have been several studies which have identified that there is a heritable aspect to the rumen metagenome, at least some of the changes to a low methane rumen should be able to be inherited, resulting on the ability to select for low methane emitting animals. This could be a useful approach for any trait where the microbiome is easier to measure than the trait itself, of which methane is a key example.

The development of metagenomic predictions to rapidly build large databases of proxy phenotypes to develop genomic breeding values would be enabled if the target microbiome is optimised for ease of sampling. In the case of enteric methane production, proxy databases could be generated using rumen metagenome samples, which are quicker and cheaper to obtain then direct phenotyping or could be obtained from saliva-based microbiomes. Tapio et al. (2016) investigated this using qPCR (quantitative polymerase chain reaction) and concluded that buccal swabs could be used as a predictor of the rumen microbiome population. The hypothesis behind this assertion is that as the animals ruminate, they deposit the rumen bacterial population in the mouth. This could provide a more user-friendly method of microbiome collection than currently available from the rumen itself.

## Conclusion

Metagenomic predictions can be used to predict the phenotype of traits that are associated with microbiome variation. Their use is still in its infancy with many areas left to explore and optimise. With large sample numbers now able to be sequenced, metagenomic predictions offer an opportunity for use as proxy traits that can take to place of challenging phenotypes that are expensive and/or difficult to measure on large numbers of individuals, such as enteric methane from ruminants. Future work should focus on dramatically increasing the size of the populations being studied. Testing new machine learning based prediction methods will become possible as the size of datasets increases. The anticipated outcome of larger populations with optimised predictions methods will be more accurate predictions that can be implemented by industry as proxy phenotypes for selection and culling.
